# Comparative Effects of Native and Defatted Flaxseeds on Intestinal Enzyme Activity and Lipid Metabolism in Rats Fed a High-Fat Diet Containing Cholic Acid

**DOI:** 10.3390/nu10091181

**Published:** 2018-08-28

**Authors:** Paulina M. Opyd, Adam Jurgoński, Jerzy Juśkiewicz, Bartosz Fotschki, Jarosław Koza

**Affiliations:** 1 Department of Biological Function of Food, Institute of Animal Reproduction and Food Research, Polish Academy of Sciences, Tuwima 10 Str., 10-748 Olsztyn, Poland; p.opyd@pan.olsztyn.pl (P.M.O.); j.juskiewicz@pan.olsztyn.pl (J.J.); b.fotschki@pan.olsztyn.pl (B.F.); 2 Department of Gastroenterology and Nutrition Disorders, Faculty of Health Sciences, Collegium Medicum in Bydgoszcz, Nicolaus Copernicus University in Toruń, Ujejskiego 75 Str., 85-168 Bydgoszcz, Poland; jaroslaw.koza@cm.umk.pl

**Keywords:** flaxseed, rat model, gastrointestinal tract, lipid metabolism

## Abstract

We hypothesize that defatting is an important factor that can determine the beneficial effects of flaxseeds on rats with diet-induced disorders. The experiment lasts 8 weeks and is conducted on Wistar rats allocated to four groups as follows: a control group fed with a standard diet; a high-fat (HF) group fed with a diet containing 21% fat and 0.1% cholic acid as a stimulator of lipid absorption; an HF group fed a diet supplemented with 1% native flaxseeds; and an HF group fed a diet supplemented with 1% defatted flaxseeds. In the HF group, several unfavourable changes in the gut and lipid metabolism are observed. Supplementation of the HF diet with native flaxseeds prevent an increase in colonic β-glucuronidase activity, whereas dietary defatted flaxseeds increase mucosal disaccharidase activities in the small intestine (sucrose, maltase and lactase). Regardless of the form of supplementation, dietary flaxseeds increase bacterial glycolytic activity in the distal intestine and decrease hepatic fat, especially triglyceride, accumulation. Both flaxseed forms decrease lipid peroxidation in the kidneys and increase the blood HDL cholesterol concentration with the native form being more efficient in the former and the defatted form being more efficient in the latter. The lipid-modulating effects of defatted flaxseeds are associated with reduced hepatic expression of peroxisome proliferator-activated receptor α, which is not the case in terms of native flaxseeds. Dietary supplementation with a relatively small amount of flaxseeds can exert beneficial effects on gut functions and lipid metabolism in rats, and these effects are affected by defatting to some extent.

## 1. Introduction

Common flax (*Linum usitatissimum* L.) is an example of a well-studied oilseed crop with a high content of nutrients and non-nutrient compounds [1]. Primarily, flaxseeds contain approximately 40% oil, which is usually extracted by cold pressing for consumption purposes [2]. Although flaxseed oil contains relatively small amounts of linoleic and oleic acid (ca. 15% and 20% of total fatty acids, respectively), it is exceptionally rich in α-linolenic acid, an essential *n*-3 fatty acid, and the proportion of which in this oil can constitute up to 57% of total fatty acids [3]. Thus, flaxseed oil is a valuable foodstuff that can help lower the ratio of *n*-6 to *n*-3 polyunsaturated fatty acids (PUFA) in the diet [4]. PUFAs in the *n*-3 family improve lipid metabolism due to their ability to induce fatty acid oxidation in the liver and skeletal muscles, as well as simultaneous suppression of hepatic lipid synthesis [5]. Moreover, *n*-3 PUFAs alter the fatty acid composition of cell membranes, thus reducing inflammation in the body. It is also thought that *n*-3 PUFAs lower blood pressure and reduce lipid accumulation in blood vessel walls, which contributes to the prevention of cardiovascular disease [6].

Flaxseeds are rich in other nutrients and biologically active compounds, especially in protein, fibre and phenolic compounds, and they are also a source of cyanogenic glycosides [7]. The protein content of flaxseed is approximately 20%, and its amino acid pattern is similar to that of soybean [3]. Flaxseed protein is rich in arginine, aspartic acid and glutamic acid, whereas lysine is the limiting amino acid [7]. Interestingly, some studies have shown that flaxseed protein hydrolysates have some potential antihypertensive [8] and antioxidative activities [9,10]. The fibre content in flaxseeds is approximately 28 g for 100 g of the seeds. Two-thirds of the fibre is insoluble, and one one-third of the fibre is soluble. However, a predominant part of the soluble fraction consists of mucilage gums with a high water-binding capacity. Thus, flaxseeds are often used as a remedy in gastrointestinal dysfunctions [7]. Mucilage affects multiple aspects of the gastrointestinal function as it retards gastric emptying, reduces apparent fat digestibility and increases bulking in the colon [11,12]. Moreover, flax mucilage also lowers cholesterolaemia and contributes to the regulation of glycaemia [13,14]. The most important phenolic compounds in flaxseeds are lignans with high in vitro antioxidant activities, mainly secoisolariciresinol diglucoside (SDG), the content of which equals up to 1 g/100 g of seeds and is even higher in defatted flaxseeds (1.8 g/100 g), indicating that they do not pass in large quantities into the oil during cold-pressing. Nevertheless, flaxseeds also contain some toxic compounds, mainly cyanogenic glycosides (approximately 400 mg for 100 g of the seeds) [15].

The consumption of flaxseeds has been shown to improve dyslipidaemia and reduce blood pressure, and flaxseeds also have some anti-inflammatory and anti-obesity actions. Interestingly, all the aforementioned beneficial effects of flaxseed have been attributed not only to PUFAs, mainly α-linolenic acid, but also to fibre and lignans [15]. Thus, the aim of this study was to compare the effects of a high-fat (HF) diet supplemented with a small amount of native or defatted flaxseeds on gastrointestinal tract, liver and kidney functions as well as lipid metabolism in rats. An HF diet rich in saturated fatty acids and supplemented with cholic acid, as a stimulator of lipid absorption, was used to induce metabolic disorders in rats. We hypothesized that the defatting of flaxseeds is an important factor that can determine their beneficial effects on rats with diet-induced disorders.

## 2. Materials and Methods

### 2.1. Preparation of Flaxseeds

The seeds of flax (*Linum usitatissimum* L.) were purchased in two forms, namely native and defatted (Oleofarm Ltd., Wrocław, Poland). Both forms were ground for 1 min prior to use as dietary supplements in the feeding experiment. The detailed composition of the seeds is shown in Table 1.

### 2.2. Animal and Diets

The feeding experiment was conducted using 32 male Wistar rats allocated to four groups with eight animals in each group. The rats were individually housed in plastic cages and controlled environment (a 12-h light-dark cycle, a temperature of 21 ± 1 °C, a relative humidity of 50% to 70% and 20% air changes per hour). The initial body weight was comparable among groups and is shown in Table 2. Each group was fed with a modified version of the semi-purified rodent diet recommended by Reeves [16]. The control (C) group was fed a standard diet that contained 7% rapeseed oil and 53% cornstarch as the sole sources of fat and digestible carbohydrates, respectively. The diet in the HF group was modified by the addition of lard and cholic acid (14% and 0.1% diet, respectively) at the expense of cornstarch. The other two groups were fed the HF diet supplemented with 1% native or defatted flaxseeds (HF + FS and HF + DFS group, respectively). The detailed composition of the diets, which were freely available to rats for the entire experimental period, is shown in Table 1. The experiment was conducted in compliance with the European guidelines for the care and use of laboratory animals, and the animal protocol employed in this study was approved by the local Institutional Animal Care and Use Committee in Olsztyn, Poland (permission number: 37/2017).

### 2.3. Collection of Biological Material and Analytical Procedures

After 8 weeks of experimental feeding, rats were anaesthetized with a mixture of xylazine and ketamine in physiological salt (10 mg and 100 mg/kg body weight, respectively). Each animal was then weighed, and the abdomen was cut open. The blood was subsequently collected from the tail vein. Serum was prepared by solidification and low-speed centrifugation (2500× *g* for 10 min at 4 °C). Select internal organs (i.e., small intestine, caecum, colon, kidneys and liver) were removed, weighed and used for further investigation.

Disaccharidase activity (lactase, maltase and sucrase) was assayed in jejunal mucosa samples according to the previously described method of Dahlqvist with modifications [17]. Briefly, an aliquot of mucosal homogenate (0.1 mL) was incubated at 37 °C with 0.1 mL of a substrate solution (sucrose, maltose or lactose) in a phosphate buffer solution (pH 7.0). After 15 min of incubation, cold distilled water was added, and the enzymatic reaction was interrupted by immersion of the test tube in boiling water for 3 min. A blank with the same composition was simultaneously prepared and immersed in boiling water without prior incubation. Released glucose was quantified using a glucose oxidase reagent (Alpha Diagnostic Ltd., Warsaw, Poland), and the disaccharidase activity was expressed as µmol of glucose liberated from disaccharide per min per gram of protein. The mucosal protein concentration was determined using the Bradford method with bovine serum albumin as the standard.

Samples of fresh ileal, caecal and colonic digesta were collected, and their pH values were measured using a microelectrode and pH/ION meter (model 301, Hanna Instruments, Amorim, Povoa de Varzim, Portugal). The small intestinal digesta was diluted with distilled water (1:1), mixed and then centrifuged at 10,000× *g* for 10 min. The supernatant (0.5 mL) was placed in a cone-plate rotational viscometer (model DV-II+; Brookfield Engineering Laboratories, Stoughton, USA), and the viscosity was measured as apparent viscosity at a fixed temperature of 37 °C and a shear rate of 60 per second. The ammonia concentration in the fresh caecal digesta was extracted, trapped in a solution of boric acid and then quantified by direct titration with sulphuric acid in Conway dishes. The concentration of short-chain fatty acids (SCFAs) was determined in caecal digesta after storage at −20 °C using gas chromatography (Shimadzu Co., Nakagyo, Kyoto, Japan) and a capillary column (SGE BP21, 30 m × 0.53 mm; SGE Europe Ltd., Milton Keynes, UK) as previously described [18]. Microbial glycolytic activity in the colonic digesta (α- and β-glucosidase; α- and β-galactosidase; and β-glucuronidase) was measured spectrophotometrically using the rate of p- or o-nitrophenol release from nitrophenylglucosides (Sigma-Aldrich, St. Louis, MO, USA) according to the method described by Barczyńska et al. [18]. The enzymatic activity was expressed as µmol of product formed per hour per gram of digesta.

The serum concentrations of cholesterol (total, HDL fraction and LDL fraction), triglycerides, urea and creatinine as well as the serum activity of aspartate transaminase (AST) and alanine transaminase (ALT) were determined using an automatic biochemical analyser (Pentra C200, Horiba Ltd., Kyoto, Japan).

Liver lipids were extracted according to the Folch method [19] with some modifications. Briefly, the liver tissue was homogenized with a 2:1 mixture of chloroform–methanol (0.2 g in 4 mL of mixture) using a high-performance homogenizer (IKA T25 digital ULTRA-TURRAX^®^, Wilmington, NC, USA) and then centrifuged at 15,000× *g* for 10 min. The supernatant was washed with 0.8 mL of distilled water, vortexed and centrifuged for 15 min (2500× *g*). After removing the upper phase, the lower phase containing lipids was evaporated under a nitrogen stream at 37 °C. Lipids were dissolved with 2.88 mL of chloroform, and cholesterol and triglycerides were determined spectrophotometrically using reagents from Alpha Diagnostics Ltd. (Warsaw, Poland). The glutathione (GSH) and glutathione disulphide (GSSG) contents in the liver tissue were determined spectrophotometrically using the method of Rahman et al. [20]. Thiobarbituric acid-reactive substances (TBARS) were determined in kidney and liver tissue after storage at −20 °C using a procedure developed by Botsoglou et al. [21]. The TBARS content was determined spectrophotometrically at 532 nm and expressed in microgram malondialdehyde per gram of tissue.

Total RNA was extracted from liver samples using the TRI Reagent (Sigma-Aldrich, St. Louis, MO, USA) according to the manufacturer’s instructions. Quantity and quality of RNA were measured spectrophotometrically using a NanoDrop1000 (Thermo Fisher Scientific, Waltham, MA, USA) and agarose gel electrophoresis, respectively. cDNA was synthesized from 500 ng of total RNA using a High-Capacity cDNA Reverse Transcription Kit with RNase Inhibitor (Applied Biosystem, Waltham, MA, USA). Glyceraldehyde 3-phosphate dehydrogenase (Gapdh) was selected as a reference gene. The levels of peroxisome proliferator-activated receptor α (PPARα), peroxisome proliferator-activated receptor γ (PPARγ) and Gapdh mRNA expression were analysed using Single Tube TaqManVR Gene Expression Assays (Life Technologies, CA, USA). Amplification was performed using a 7900HT Fast Real-Time PCR System under the following conditions: initial denaturation for 10 min at 95 °C; 40 cycles of 15 s at 95 °C and 1 min at 60 °C. Each run included a standard curve based on aliquots of pooled liver RNA. All samples were analysed in duplicates. mRNA expression levels of PPARα and PPARγ were normalized to Gapdh and multiplied by 10.

### 2.4. Statistical Analysis

Values are expressed as the mean ± standard error of the mean (SEM, *n* = 8). Data were tested by one-way analysis of variance and Duncan’s multiple range posthoc tests using STATISTICA version 12 (StatSoft Corp., Kraków, Poland). A difference of *p* ≤ 0.05 was considered statistically significant.

## 3. Results

The effects of dietary supplementation with native or defatted flaxseeds on rat body weight and physiological indices of the gastrointestinal tract are shown in Table 2. After eight weeks of experimental feeding, the body weight was higher in the HF and HF + DFS groups than in the C group, whereas dietary supplementation with native flaxseeds (group HF + FS) slightly decreased the body weight to a level comparable with that of the C group. As compared to the C group, the mass of small intestine with digesta significantly increased in the HF + FS group. The pH value of ileal digesta was decreased in all HF groups compared to the C group. Sucrase, maltase and lactase activities in the small intestinal mucosa were increased in the HF + DFS group compared to the C and HF groups. Neither the HF diet nor the tested types of flaxseeds had an influence on selected indices of the caecum, including the mass, pH value of digesta, ammonia concentration and SCFA concentration in the digesta. The colonic digesta mass was significantly lower in the HF + FS group than in the other groups. The HF diet feeding decreased the α-glucosidase and α-galactosidase activity in the colonic digesta (HF group). In the HF + FS and HF + DFS groups, the activities of β-glucosidase and β-galactosidase were significantly higher compared to those of the C and HF groups. The β-glucuronidase activity was significantly increased in the HF and HF + DFS groups compared to those in the C and HF + FS groups.

Markers of liver and kidney functions are shown in Table 3. After eight weeks of experimental feeding, the relative liver mass did not differ among groups, whereas the liver fat content was significantly higher in the HF group compared to that in the C group. In the HF + FS and HF + DFS groups, the liver fat content was slightly decreased to a level comparable to that of the C group. The liver cholesterol content was significantly increased in all HF groups compared to the C group. The liver triglyceride content was significantly increased in the HF group, whereas flaxseeds, regardless of the supplementation form, significantly reduced the liver triglyceride content but not to the level of group C. The serum ALT activity was not influenced by experimental feeding, whereas the serum AST activity was significantly increased after native flaxseed supplementation. The type of diet had no influence on the GSH/GSSG ratio and TBARS concentration in the liver. Moreover, the HF diet caused a significant reduction of kidney mass relative to body weight. Dietary supplementation with flaxseeds caused an increase in the relative kidney mass, but the increase was only significant in the HF + DFS group. The serum concentration of creatinine and urea was not affected by the dietary treatments, whereas native flaxseeds markedly decreased the kidney TBARS content (HF + FS group vs. C and HF groups).

The effects of dietary native and defatted flaxseed supplementation on the blood lipid profile are shown in Table 4. The total cholesterol, LDL cholesterol and triglyceride concentrations were comparable among all groups. The HDL cholesterol concentration was significantly decreased in the HF group compared to the C group. Dietary supplementation with defatted flaxseed significantly increased the HDL cholesterol concentration to a level comparable with that of the C group, whereas the supplementation with native flaxseeds caused only a slight increase in HDL cholesterol.

**Figure 1:**
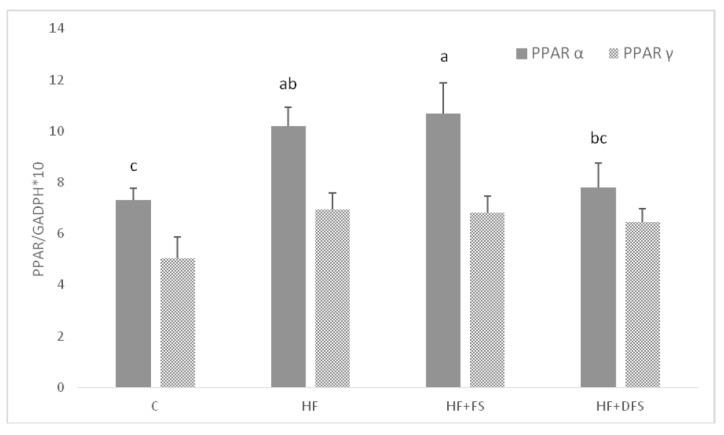
qRT-PCR analysis of PPARα and PPARγ mRNA expression in rat livers. All values are expressed as the mean ± SEM (*n* = 8). Values not sharing the same superscript (^a,b,c^) within a row are different at *p* ≤ 0.05. C: group fed a standard diet for laboratory rodents; HF: group fed a high saturated fat-containing diet; HF + FS: group fed a high saturated fat-containing diet supplemented with native flaxseeds; HF + DFS: group fed a high saturated fat-containing diet supplemented with defatted flaxseeds; PPARα: peroxisome proliferation-activated receptor α; PPARγ: peroxisome proliferation-activated receptor γ; GAPDH: glyceraldehyde 3-phosphate dehydrogenase (housekeeping gene); qRT-PCR: quantitative reverse transcriptase real-time polymerase chain reaction.

The effects of dietary native and defatted flaxseed supplementation on PPARα and PPARγ mRNA expression in rat livers are shown in Figure 1. PPARγ expression was comparable among all groups, whereas PPARα expression was significantly increased in the HF and HF + FS groups compared to the C group. Dietary addition of defatted flaxseeds decreased PPARα expression to the level found in the C group.

## 4. Discussion

Flaxseeds are present in the market in various types of products both in native and defatted forms [15,22]. The present study was conducted to compare the health-related effects of these two flaxseed forms on gastrointestinal tract, liver and kidney functions as well as lipid metabolism in rats with disorders induced by an HF diet containing cholic acid. Native flaxseeds were more caloric and contained approximately 5 times more fat than their defatted form (details in Table 1). In contrast, the defatted flaxseed preparation contained more protein and almost twice the amount of fibre. Both forms were added to the HF diet at the expense of cornstarch and in the same relatively low amount (1% of the HF diet). This amount simulated the daily consumption of flaxseeds by humans, and the caloric value and nutrient content of the diets remained comparable, which allowed for a simple and feasible comparison of both flaxseed types. However, native flaxseeds are usually consumed as a whole, whereas both forms were ground in this study before adding them to the diet, which was done to standardize the material and diets.

Feeding with an HF diet is the basic way to obtain obesity and disorders related to lipid metabolism in rats [23]. Cholic acid was additionally included in the HF diet as a stimulator of lipid absorption to intensify metabolic disorders in the rats. After 8 weeks of experimental feeding, a significantly higher body weight was observed in the HF group compared to the C group. The HF diet also increased hepatic cholesterol and triglyceride levels by approximately four and two times, respectively (compared with the C group). Studies on HF diets, including those conducted in our laboratory, have suggested that such a considerable hepatic cholesterol accumulation cannot be achieved in rats by only a high dietary fat content [24,25]. For example, the study by Quesada et al. [24] showed that the cholesterol accumulation in rats fed an HF diet for 12 weeks, although significantly higher, is not even 2 times greater. Thus, exogenous cholic acid provided with an HF diet is an important factor, leading to hepatic cholesterol accumulation in rats. Of note, cholic acid is one of two primary bile acids synthesized by the human liver, which facilitates digestion and absorption of dietary lipids [26]. However, this compound is minor in rats, in which muricholic acids are the main bile acids synthesized by the liver [25]. Interestingly, the HF diet containing cholic acid used in the present study had minor, but still negative influence on the blood lipid profile, decreasing only the HDL cholesterol concentration. As a comparison, a previous study by our laboratory using an HF diet without the inclusion of cholic acid showed increases in the total cholesterol in rat blood samples [27].

Examples of preparations that contain either native or defatted flaxseeds are used for alleviating gastrointestinal dysfunctions. In the present study, both flaxseed forms affected the intestinal functions of rats but in a slightly different manner. Specifically, the addition of defatted flaxseeds, but not native seeds, to the HF diet had stimulatory effects on disaccharidase activity in the small intestinal mucosa (sucrose, maltase and lactase). However, it is difficult to speculate the reason for this difference. Perhaps the higher content of carbohydrates and dietary fibre played a role, suggesting that some specific saccharides in defatted flaxseeds played a role. Moreover, both native and defatted flaxseed supplementation increased bacterial glycolytic activity in the colonic digesta (α- and β-glucosidase and galactosidase activity). A soluble fibre fraction of flaxseeds, mainly mucilage, may have been responsible for these changes. Mucilage consists of two polysaccharide fractions as follows: a neutral arabinoxylan and an acidic pectic-like polymer [28]. A confirmation of this supposition can be the study of Alzueta et al. [29], who reported that dietary flaxseeds, but not their demucilaged form, stimulate the intestinal microbiota of broiler chickens. Another factor that can affect bacterial enzyme activity is SDG because it is fermented by colonic bacteria to form enterolactone and enterodiol [15]. In the present study, the HF diet increased colonic β-glucuronidase activity, whereas the native flaxseed supplementation prevented that increase. Generally, the suppression of bacterial β-glucuronidase activity is considered beneficial for the body because this enzyme releases some toxic or even carcinogenic substances that are excreted together with bile to the intestinal tract [30].

The kidney functions of rats were not considerably affected either by the HF diet or by dietary flaxseeds (regardless of type) as indicated by the serum creatinine and urea levels (Table 3). Nevertheless, the HF diet reduced the relative kidney mass, whereas dietary flaxseeds, especially in their defatted form, prevented that decrease. A similar unfavourable effect of the HF diet has been observed in an earlier study from our laboratory [27], but the reason for the decreased kidney mass remains unknown. Moreover, dietary flaxseeds, especially their native form, decreased the TBARS content in the kidney, which is a marker of lipid peroxidation. Kaur et al. [31] suggested a high correlation between the antioxidant capacity and the total phenolic content of flaxseeds, which indicates that the latter may have been responsible for the beneficial decrease of kidney TBARS content in the present study. Interestingly, Alu’datt et al. [32] showed that the full-fat meal from flaxseeds has significantly higher antioxidant activity compared to the defatted meal, which was in accordance with the present results.

In the present study, the HF diet feeding increased PPARα mRNA expression in the rat livers. This result agreed with the study by Patsouris et al. [33], who showed that PPARα expression and PPARα signalling are activated in the liver by chronic HF feeding. PPARα is a specific transcription factor involved in the metabolism of fatty acids [5], and it directly up-regulates genes coding proteins involved in fatty acid uptake and β-oxidation [5,34]. HF consumption increases the amount of fatty acids arriving to the liver and, thus, the requirement for hepatic fatty acid uptake and oxidation. In the present study, however, despite the up-regulation of PPARα by the HF diet, negative changes resulting from the excess fat supply, i.e., hepatic accumulation of cholesterol and triglycerides, were still observed, suggesting that the up-regulation of PPARα was not sufficient to efficiently resolve the extra load of fat. Nevertheless, dietary supplementation with defatted flaxseeds slightly decreased PPARα expression, which was partly in accordance with decreased hepatic fat, especially triglyceride accumulation, in both flaxseed groups. However, other factors were also apparently involved in these beneficial decreases. Interestingly, Sun et al. [35] suggested that flaxseed SDG is responsible for the reduction of hepatic fat and triglyceride accumulation. Moreover, dietary flaxseeds, especially defatted flaxseeds, elevated the serum HDL cholesterol concentration, which was significantly decreased by the HF diet. This result corroborated with previous reports demonstrating a similar beneficial effect of flaxseeds on blood cholesterolaemia [36,37]. The increase in the HDL cholesterol level can be attributed to the flaxseed fibre because soluble fibres regulate the blood cholesterol profile by the following three possible mechanisms: prevention of bile salts re-absorption and their excess faecal excretion; reduction of glycaemic response, thus lowering insulin stimulation of hepatic cholesterol synthesis; and fermentation of soluble fibres by intestinal microbiota, which produces propionate, thereby inhibiting cholesterol synthesis [38,39]. Interestingly, the effect was especially pronounced in the HF + DFS group, which was probably due to the higher content of fibre in defatted seeds. Some authors have also suggested a role of lignans in increasing the HDL cholesterol concentration, but their mechanism of action has not been recognized thus far [40].

The main limitation of this study was that although flaxseeds were defatted, they still contained some amounts of fat. Thus, precise assessing to which extent the oily fraction of flaxseeds was responsible for their beneficial effects was not possible. This issue should be addressed in future research, in which adequately composed diets containing either flaxseeds or an oil obtained from them have to be applied in order to compare their beneficial effects.

## 5. Conclusions

In conclusion, an HF diet containing cholic acid leads to a number of unfavourable changes in the gastrointestinal tract and lipid metabolism of rats, such as colonic disruption of bacterial enzyme activities or increase in the accumulation of lipids in the liver, especially cholesterol and also triglycerides, together with the increase in the PPARα expression. Dietary supplementation with a relatively small amount of flaxseeds can exert beneficial effects on intestinal tract functions and lipid metabolism in rats, which are, to some extent, affected by defatting. Dietary supplementation with native flaxseeds prevented the increase of colonic β-glucuronidase activity, whereas dietary defatted flaxseeds increased mucosal disaccharidase activities in the small intestine. Regardless of the form of supplementation, dietary flaxseeds increased bacterial glycolytic activity in the distal intestine and decreased hepatic fat accumulation, especially triglyceride accumulation. Both flaxseed forms decreased lipid peroxidation in the kidneys and increased the blood HDL cholesterol concentration. However, the native flaxseed form was more efficient in the former, whereas the defatted flaxseed form was more efficient in the latter. The lipid-modulating effects of defatted flaxseeds, but not of native flaxseeds, was associated with a reduced hepatic expression of PPARα. Overall, these findings indicated that both native and defatted flaxseeds are a valuable dietary factor for the prevention and treatment of diet-related metabolic disorders.

## Figures and Tables

**Table 1:** Composition of diets fed to rats (%).

Diet Components	C	HF	HF + FS	HF + DFS
Casein	20	20	20	20
dl-methionine	0.3	0.3	0.3	0.3
Rapeseed oil	7	7	7	7
Lard	-	14	14	14
Cholic acid	-	0.1	0.1	0.1
Corn starch	53.0	38.9	37.9	37.9
Flaxseeds ^1^	-	-	1	-
Defatted flaxseeds ^2^	-	-	-	1
Saccharose	10	10	10	10
Cellulose	5	5	5	5
Mineral mix ^3^	3.5	3.5	3.5	3.5
Vitamin mix ^3^	1	1	1	1
Choline chloride	0.2	0.2	0.2	0.2

^1^ Composition (per 100 g of product): kilocalories, 544; fat, 46 g (saturated fatty acids, 5.2 g; monounsaturated fatty acids, 8.8 g; polyunsaturated fatty acids, 32.0 g); carbohydrates, 2 g; protein, 20 g; dietary fibre, 20 g. ^2^ Composition (per 100 g of product): kilocalories, 311; fat, 9 g (saturated fatty acids, 1.1 g; monounsaturated fatty acids, 1.7 g; polyunsaturated fatty acids, 6.2 g); carbohydrates, 7 g; protein, 32 g; dietary fibre, 38 g. ^3^ Recommended for the AIN-93 diet [16]. C: group fed a standard diet for laboratory rodents; HF: group fed a high saturated fat-containing diet; HF + FS: group fed a high saturated fat-containing diet supplemented with native flaxseeds; HF + DFS: group fed a high saturated fat-containing diet supplemented with defatted flaxseeds.

**Table 2:** Body weight and physiological indices of the gastrointestinal tract of rats.

Indices	C	HF	HF + FS	HF + DFS	ANOVA *p* Value
Initial BW, g	290 ± 2.2	296 ± 2.5	295 ± 2.2	295 ± 3.0	NS
Final BW, g	404 ± 5.1 ^b^	429 ± 5.4 ^a^	423 ± 8.8 ^a,b^	429 ± 6.1 ^a^	<0.05
Small intestine					
Mass with digesta, g/100 g BW	1.44 ± 0.025 ^b^	1.52 ± 0.044 ^a,b^	1.62 ± 0.041 ^a^	1.52 ± 0.035 ^a,b^	<0.05
pH of digesta	7.31 ± 0.134 ^a^	6.85 ± 0.088 ^b^	6.90 ± 0.107 ^b^	6.72 ± 0.133 ^b^	<0.01
Digesta viscosity, mPa*s	2.01 ± 0.169	1.91 ± 0.130	1.74 ± 0.133	1.63 ± 0.188	NS
Mucosal disaccharidase activity ^1^					
Sucrase	18.5 ± 2.46 ^b^	23.3 ± 3.45 ^b^	24.5 ± 3.84 ^a,b^	35.8 ± 5.31 ^a^	<0.05
Maltase	81.0 ± 12.6 ^b^	102.6 ± 15.7 ^b^	105.4 ± 16.0 ^b^	158.3 ± 22.1 ^a^	<0.05
Lactase	2.95 ± 0.581 ^b^	3.03 ± 0.433 ^b^	3.58 ± 0.442 ^b^	5.47 ± 0.500 ^a^	<0.01
Caecum					
Tissue mass, g/100 g BW	0.155 ± 0.004	0.141 ± 0.008	0.151 ± 0.003	0.159 ± 0.005	NS
Digesta mass, g/100 g tissue	1.69 ± 0.156	1.62 ± 0.152	1.40 ± 0.224	1.23 ± 0.151	NS
pH of digesta	7.45 ± 0.091	7.56 ± 0.073	7.57 ± 0.109	7.41 ± 0.100	NS
Ammonia, mg/g digesta	0.314 ± 0.027	0.366 ± 0.050	0.328 ± 0.022	0.340 ± 0.019	NS
SCFA concentration ^2^	65.7 ± 3.89	65.8 ± 3.35	64.6 ± 5.68	62.3 ± 3.78	NS
Colon					
Tissue mass, g/100 g BW	0.259 ± 0.019	0.262 ± 0.017	0.253 ± 0.013	0.232 ± 0.009	NS
Digesta mass, g/100 g tissue	0.835 ± 0.122 ^a^	0.785 ± 0.124 ^a^	0.469 ± 0.073 ^b^	0.642 ± 0.043 ^a^	<0.05
pH of digesta	7.23 ± 0.069	7.21 ± 0.114	7.22 ± 0.097	7.02 ± 0.088	NS
Digesta bacterial enzymes activity ^3^					
α-glucosidase	31.1 ± 2.73 ^a^	21.9 ± 1.19 ^b^	29.4 ± 1.78 ^a^	28.2 ± 1.78 ^a^	<0.05
β-glucosidase	6.79 ± 0.77 ^b^	3.65 ± 0.54 ^b^	10.10 ± 0.98 ^a^	13.26 ± 1.70 ^a^	<0.001
α-galactosidase	22.7 ± 1.34 ^a^	15.6 ± 1.15 ^b^	25.3 ± 2.09 ^a^	21.7 ± 1.62 ^a^	<0.01
β-galactosidase	100 ± 10.3 ^b^	92 ± 3.0 ^b^	133 ± 10.1 ^a^	129 ± 7.7 ^a^	<0.01
β-glucuronidase	62.2 ± 7.72 ^b^	91.2 ± 4.97 ^a^	63.4 ± 6.18 ^b^	88.7 ± 12.29 ^a^	<0.05

All values are expressed as the mean ± SEM (*n* = 8). Values not sharing the same superscript (^a,b^) within a row are different at *p* ≤ 0.05. C: group fed a standard diet for laboratory rodents; HF: group fed a high saturated fat-containing diet; HF + FS: group fed a high saturated fat-containing diet supplemented with native flaxseeds; HF + DFS: group fed a high saturated fat-containing diet supplemented with defatted flaxseeds; BW: body weight; SCFA: short-chain fatty acid; PSCFA: putrefaction short-chain fatty acid; NS: non-significant. ^1^ µmol/min/g protein. ^2^ µmol/g digesta. ^3^ µmol/h/g digesta.

**Table 3:** Markers of liver and kidney functions in rats.

Indices	C	HF	HF + FS	HF + DFS	ANOVA *p* Value
Liver markers					
Liver mass, g/100 g BW	2.31 ± 0.029	2.47 ± 0.064	2.73 ± 0.166	2.66 ± 0.196	NS
Liver fat, %	8.09 ± 0.476 ^b^	13.85 ± 1.753 ^a^	12.10 ± 1.900 ^a,b^	12.41 ± 1.150 ^a,b^	<0.05
Liver cholesterol, mg/g	3.86 ± 0.452 ^b^	14.37 ± 0.749 ^a^	13.14 ± 0.736 ^a^	13.63 ± 1.036 ^a^	<0.001
Liver triglycerides, mg/g	25.8 ± 1.42 ^c^	50.7 ± 3.22 ^a^	40.2 ± 3.21 ^b^	40.0 ± 3.02 ^b^	<0.001
Serum ALT, U/L	93.2 ± 5.83	108.0 ± 5.42	115.1 ± 10.43	90.9 ± 6.34	NS
Serum AST, U/L	20.1 ± 1.56 ^b^	32.1 ± 5.14 ^b^	43.3 ± 8.18 ^a^	28.2 ± 3.90 ^b^	<0.05
Liver TBARS, µg/g	2.33 ± 0.178	2.69 ± 0.311	2.80 ± 0.369	2.44 ± 0.143	NS
Liver GSH/GSSG, µmol/g	9.68 ± 0.485	10.74 ± 0.354	11.95 ± 1.209	10.35 ± 1.185	NS
Kidney markers					
Kidneys mass, g/100 g BW	0.516 ± 0.011 ^a^	0.454 ± 0.011 ^b^	0.492 ± 0.019 ^a,b^	0.505 ± 0.016 ^a^	<0.05
Serum creatinine, µmol/L	28.5 ± 4.32	26.1 ± 3.39	27.5 ± 3.76	25.7 ± 2.56	NS
Serum urea, mmol/L	5.92 ± 0.307	5.62 ± 0.298	6.24 ± 0.167	5.45 ± 0.325	NS
Kidney TBARS, µg/g	3.51 ± 0.333 ^a^	3.65 ± 0.371 ^a^	2.43 ± 0.428 ^b^	2.66 ± 0.226 ^a,b^	<0.05

All values are expressed as the mean ± SEM (*n* = 8). Values not sharing the same superscript (^a,b,c^) within a row are different at *p* ≤ 0.05. C: group fed a standard diet for laboratory rodents; HF: group fed a high saturated fat-containing diet; HF + FS: group fed a high saturated fat-containing diet supplemented with native flaxseeds; HF + DFS: group fed a high saturated fat-containing diet supplemented with defatted flaxseeds; BW: body weight; TBARS: thiobarbituric acid-reacting substances; GSH/GSSG: glutathione to glutathione disulphide ratio; NS: non-significant.

**Table 4:** Blood lipid profile in rats.

Indices	C	HF	HF + FS	HF + DFS	ANOVA *p* Value
Total cholesterol, mmol/L	1.90 ± 0.109	1.88 ± 0.126	1.91 ± 0.090	1.83 ± 0.073	NS
HDL cholesterol, mmol/L	0.615 ± 0.027 ^a^	0.434 ± 0.042 ^b^	0.521 ± 0.029 ^a,b^	0.534 ± 0.025 ^a^	<0.01
LDL cholesterol, mmol/L	0.129 ± 0.013	0.180 ± 0.016	0.159 ± 0.026	0.163 ± 0.009	NS
Triglycerides, mmol/L	0.848 ± 0.115	0.736 ± 0.077	0.740 ± 0.060	0.766 ± 0.060	NS

All values are expressed as the mean ± SEM (*n* = 8). Values not sharing the same superscript (^a,b^) within a row are different at *p* ≤ 0.05. C: group fed a standard diet for laboratory rodents; HF: group fed a high saturated fat-containing diet; HF + FS: group fed a high saturated fat-containing diet supplemented with native flaxseeds; HF + DFS: group fed a high saturated fat-containing diet supplemented with defatted flaxseeds; ALT: serum activity of alanine transaminase; AST: serum activity of aspartate transaminase; NS: non-significant.
